# CD103-CD23+ classical hair cell leukemia: A case report and review of the literature

**DOI:** 10.1097/MD.0000000000028262

**Published:** 2021-12-23

**Authors:** Hong-li Zhao, Hong-hua Cui, Li-fang Jin, Meng Zhao, Wei-zhang Shen

**Affiliations:** Department of Hematology, The Second Hospital of Jilin University, Changchun, China.

**Keywords:** cladribine, differential, hairy cell leukaemia, immunophenotype

## Abstract

**Introduction:**

This case report is presented to improve our understanding of the atypical immunophenotype of hairy cell leukemia.

**Patient concerns:**

A 58-year-old woman presented to our department with fatigue for >10 days.

**Diagnosis:**

The patient was diagnosed with an increased proportion of abnormal lymphocytes in peripheral blood and bone marrow smear, positive for CD11c, CD19, CD20, CD22, CD25, CD123, CD200, and Kappa, partial expression of CD23, but no expression of CD103, positive for BRAF V600E mutation.

**Interventions and outcomes:**

Cladribine combined with rituximab achieved complete remission of minor residual disease negativity.

**Conclusion:**

Hairy cell leukemia is rare, and the diagnosis and differential diagnosis should be made by combining the patient's medical history, clinical manifestations, immunophenotype, gene detection, and other means. Purine nucleoside analogs are the first-line treatments.

## 1 Introduction

Recognized as an independent disease entity by the World Health Organization (WHO2008) and the revised classification of lymphoid neoplasms in 2016, hairy cell leukemia (HCL) is a clinically rare type of leukemia. The HCL immunophenotype was characterized by strong expression of CD19, CD20, CD22, and CD200. CD5, CD23, CD10, and CD27 in hair cells are usually negative or weakly expressed, whereas CD11c, CD103, CD123, and CD25 are positive.^[1]^ One case of HCL with atypical immunophenotype of CD103 (−) and CD23 (+) admitted to our department was recently reported to discuss the clinical characteristics, diagnosis, differential diagnosis, treatment, and prognosis of HCL, and to review the literature.

## 2 Case presentation

A 58-year-old woman presented to our department on November 30, 2020, due to fatigue for >10 days. The patient had no significant medical history. Physical examination findings were anemia appearance, no yellow stain on sclera, no petechiae on skin and mucous membrane, no enlargement of superficial lymph nodes of the body, no touch of liver and spleen under ribs.

**Figure 1:**
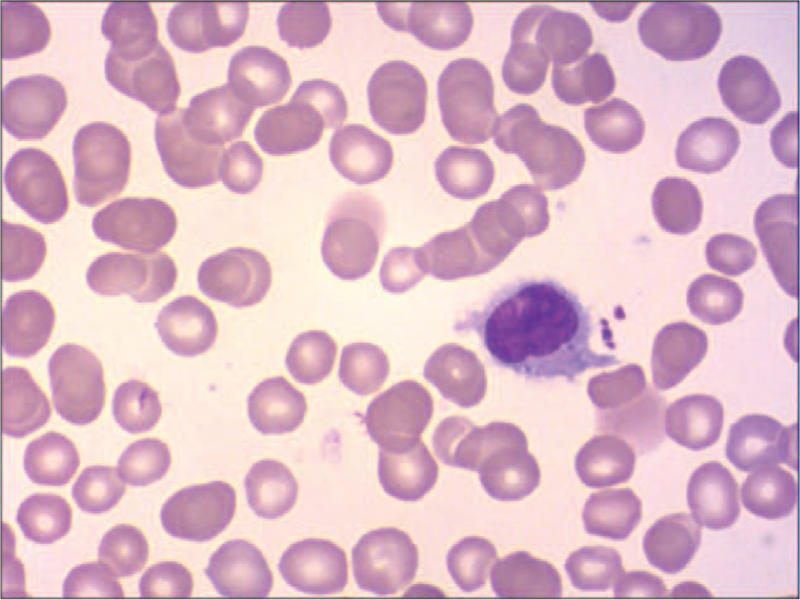
Bone marrow smear (Reischer-jimsa staining, ×1000).

**Figure 2:**
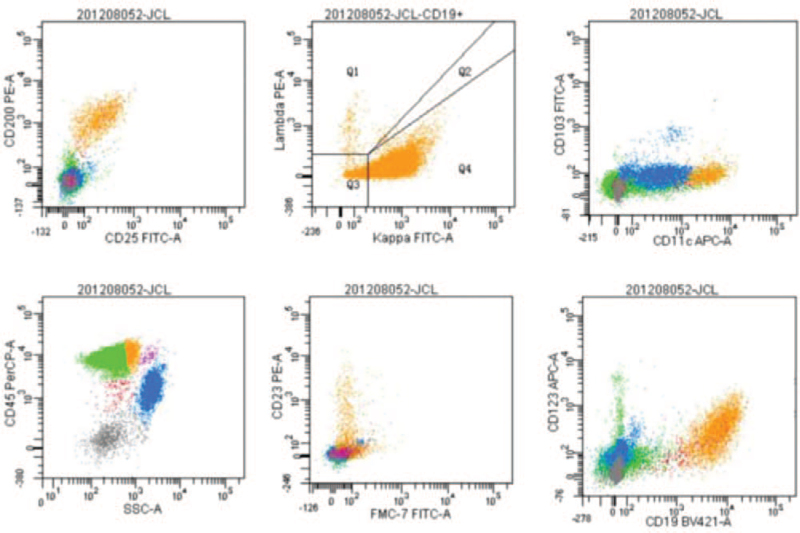
Flow cytometry immunophenotype of bone marrow in this HCL patient (CD45/SSC gating technique, ×10,000 cells).

**Figure 3:**
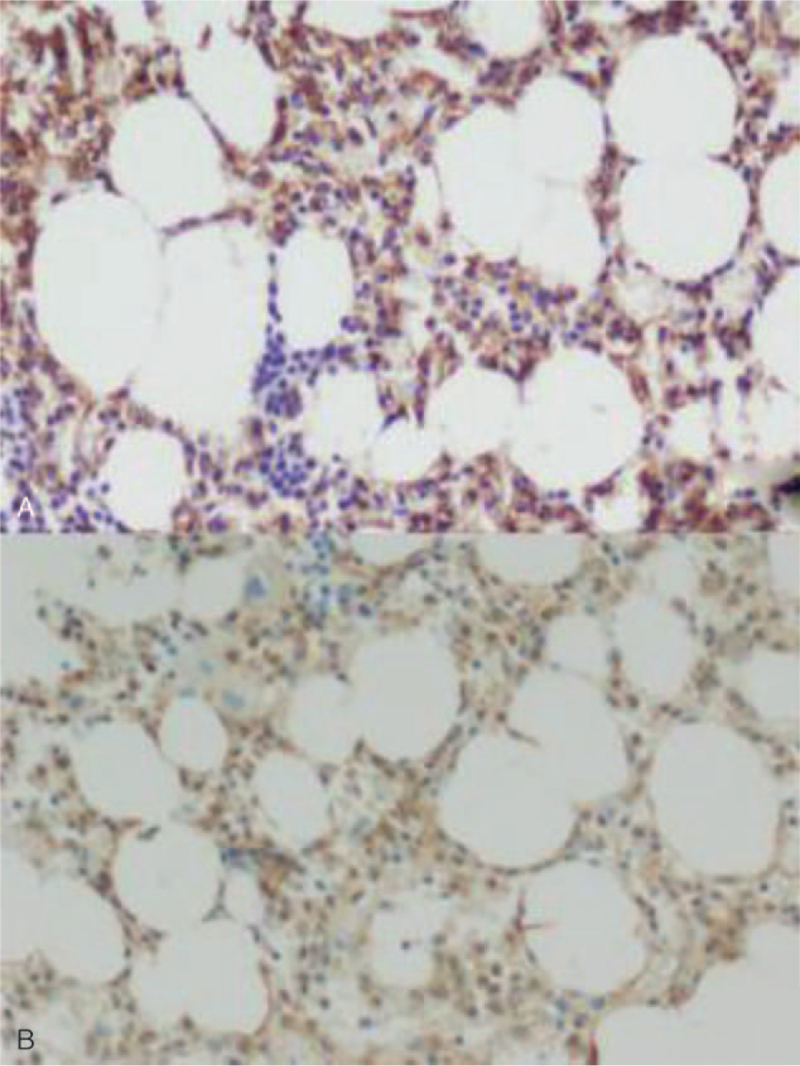
Bone marrow immunohistochemical CD20 and BRAF V600E positive (immunohistochemical staining, ×100). (A) B cell marker CD20-positive; (B) BRAF V600E positive.

**Figure 4:**
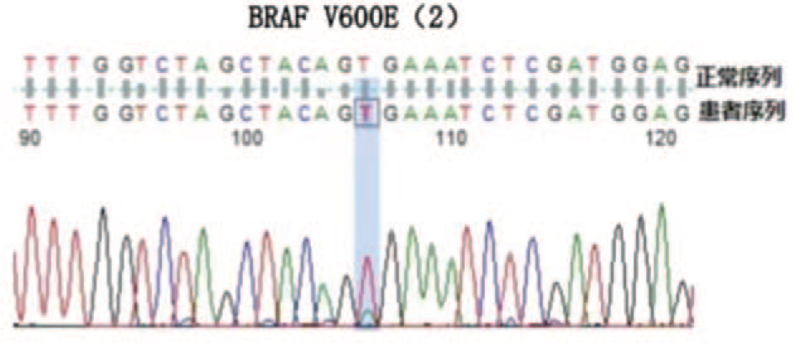
Sequencing of *BRAF* gene exon 15 in bone marrow cells of CL patients (detection methods: PCR, gene sequencing; detection content: *BRAF* gene exon 15).

Routine blood examination revealed a white blood cell (WBC) count of 1.5 × 10^9^ cells/L, neutrophil count of 30.6%, lymphocyte count of 61.0%, monocyte count of 0.1%, red blood cell count of 2.81 × 10^12^ cells/L, hemoglobin (of) 95 g/L, platelet count of 68 × 10^9^ cells/L. Lymphocytes accounted for 62% of the blood smears and 23% of the bone marrow smears. Abnormal cells accounted for 64% of bone marrow smears, with different cell sizes, large amounts of cytoplasm, gray blue, and hairy processes (Fig. 1). Bone marrow flow cytometry immunophenotyping on December 3^rd^ findings: Lymphocytes accounted for approximately 61.6% of nuclear cells, and the proportion increased. CD19+ cells accounted for approximately 14.4% of nuclear cells, expressing CD19, CD20, CD22, cKappa, Kappa, and FMC-7, partially expressing CD11c, CD23, and CD25, but not CD5, CD10. Bone marrow flow cytometry immunophenotyping was performed again on December 9: Lymphocytes accounted for about 61.8% of nuclear cells, among which CD19+ cells accounted for approximately 13.7% of nuclear cells, expressing HLA-DR, CD11c, CD19, CD20, CD22, CD25, CD123, CD200, Kappa, partially expressing CD23, but not CD5 and CD10. The 2 flow cytometric immunophenotypes were CD103− and CD23+ (Fig. 2). Bone marrow pathology revealed proliferative lymphoid cells in the bone marrow tissue. Immunohistochemical staining showed a positive B cell marker CD20 (Fig. 3A) and positive BRAFV600E (Fig. 3B). Mutation analysis of the BRAF gene showed that the missense mutation C.1799T > (P.v600E) in exon 15 of the *BRAF* gene (Fig. 4). Abdominal color Doppler ultrasonography findings: The thickness of the spleen was 4.1 cm, the subcostal diameter was 2.1 cm, and the length of the spleen was approximately 14.3 cm. Positron emission tomography-computed tomography showed that the spleen was significantly enlarged and glucose metabolism was significantly increased. The maximum standardized uptake value was 5.14. Multiple lymph nodes with increased glucose metabolism were found in the bilateral clavicular area, adjacent to the upper mediastinum trachea, adjacent to the retroperitoneal abdominal aorta, and adjacent to the vena cava. The larger one was about 12.5 × 10.5 mm in size, and the maximum standardized uptake value value was 4.20, with unclear boundaries. The patient was diagnosed with hair cell leukemia based on clinical manifestations, hair cell morphological characteristics, immunophenotype, and bone marrow pathology immunohistochemistry (BRAFV600E positive) and BRAF mutation-positive. On December 18, 2020, the treatment regimen (clatribin + rituximab) was administered, with rituximab 375 mg/m^2^, once a week, for 8 weeks; clatribin 5 mg/m2, D1-D5. The blood count began to recover after a brief decline at the beginning of the treatment. Blood routine on day 20 fingings: white blood cell 4.4 × 10^9^ cells/L, neutrophils 77.7%, lymphocytes 10.6%, monocytes 9.9%, red blood cell 2.90 × 10^12^ cells/L, hemoglobin 101 g/L, platelets 206.0 × 10^9^ cells/L. On day 48, the bone marrow smear showed no hair cells, and the bone marrow flow cytometry test was negative for minor residual disease (MRD). The patient's symptoms disappeared, and his spleen returned to normal size and was in good condition.

## 3 Discussion and literature review

HCL was first reported in 1958 as a malignant tumor with pancytopenia and splenomegaly.^[2]^ It is recognized as a separate disease entity by the World Health Organization (WHO2008) and the 2016 Revised World Health Organization Classification of Lymphatic neoplasms.^[3,4]^

HCL is a rare chronic b-lymphocyte proliferative disease, accounting for about 2% of all leukemias, with a lower incidence in Asians than in Europeans and Americans. Bone marrow is the most common organ involved and is prone to peripheral blood and spleen invasion. HCL presents an indolent course of disease, with insidious onset and unknown etiology. There were more males than females, and the male to female ratio was about 4:1. The incidence was mainly in the elderly, with a median age of 63 years, and was rare in the young. The disease is often characterized by pancytopenia, especially monocytopenia, anemia, and splenomegaly. Liver and superficial lymph node enlargement is less common. Blood smear and bone marrow smear were visible to the “hair cell.” This type of cell is small B cells, slightly larger than the normal size of lymphocytes. The nucleus was round or oval, chromatin was rough, cytoplasm was rich, cytoplasm was gray-blue, and there were pseudopod-like hairy protrusions. Bone marrow pathology can show characteristic fried egg-like changes. Bone marrow puncture is often “dry aspiration” and reticular fibrous hyperplasia can be seen on biopsy.^[5]^ However, in China, the proportion of pancytopenia, bone marrow dry aspiration, and combined myelofibrosis in HCL patients is relatively low.^[6]^

The morphology of hair cells in the peripheral blood or bone marrow of patients is an important indication and basis for the diagnosis of the disease. However, the proportion of hair cells varies and not all are typical. It is not a specific manifestation of HCL and needs to be differentiated from other HCL-like diseases, including variant hair cell leukemia (HCL-V), splenic marginal zone lymphoma (SMZL), and diffuse splenic red myeloid small B-cell lymphoma. These 4 types of diseases are chronic B-cell proliferative diseases, and villous cells can be seen in the bone marrow and peripheral blood. They showed common clinical characteristics:^[5]^ 1. The disease progresses slowly and can be relieved after treatment; however, it is difficult to cure and can be transformed into an invasive disease. 2. It is more common in middle-aged and elderly individuals. 3. All expressed mature B cell-associated antigens, but CD5 and CD10 were negative. 4. Most of the heteromorphic cells were small to medium sized lymphocytes. HCL has specific immunophenotype and molecular markers. In immunophenotype, HCL cells were positive for CD11c, CD25, CD103 and CD123 in addition to expressing mature B cell associated antigens. CD23, CD5, and CD10 are usually negative.^[1]^ An immune score system was established according to whether CD11c, CD103, CD123, and CD25 were expressed, and each expression marker was 1 point. 98% of HCL immunity scores were 3 or 4, whereas other HCL-like diseases were 0 or 1.^[7]^ BRAF V600E has a high mutation rate in classical HCL and is a molecular marker for the diagnosis of HCL.^[1]^ Tiacci et al conducted parallel sequencing of the entire exome of 242 patients with leukemia.^[8]^ BRAF V600E mutation was found in 47 patients with HCL. None of the 195 other patients with peripheral B-cell lymphoma or leukemia carried the BRAFV600E mutation, including 38 patients with splenic marginal zone lymphoma or splenic B-cell lymphoma/leukemia, which were not classified. In 2008, the WHO non-classifiable splenic B-cell lymphoma/leukemia included diffuse splenic red myeloid small B-cell lymphoma (SDRPSBCL) and HCL-V.^[3]^

HCL-V is a rare proliferative disease in mature B cells. It is characterized by spleen enlargement and “hair cells” in the peripheral blood and bone marrow. Compared with HCL, HCL-V is a more aggressive disease. The median survival time was shorter, and the response to purine analogs was worse. HCL-V has increased peripheral blood lymphocytes, including CD20 (+), CD22 (+), CD25 (−), CD123 (−), CD11c (−), CD103 (+/−), and BRAF V600E mutation (−).^[9]^

SMZL accounts for <2% of all lymphatic system malignancies. The age of onset was >60 years, and the incidence was the same in both men and women. The main manifestation is splenomegaly, and most patients may involve the peripheral blood and bone marrow. There are no characteristic molecular markers, and approximately 30% of SMZLs have KLF2 mutations.^[10]^ Expression of mature B cell surface antigen, may express DBA44, CD38, CD23(−), CD25(−), CD103 (−), CD123 (−), and CD11c positive rate is about 50%.^[10,11]^

In 2008, the WHO established SDRPSBCL as an independent disease entity,^[3]^ which belongs to splenic B-cell lymphoma/leukemia, not classified. SDRPSBCL affects people >40 years of age, mainly involving the spleen, bone marrow, and peripheral blood. Leukopenia and thrombocytopenia may be present, and splenomegaly is present in all patients, with occasional skin manifestations. Its immunophenotype is CD20 (+), CD23 (−), CD25 (−), CD123 (−), CD103 (−), and CD11c positive rate is about 87%.^[10]^ About 24% of SDRPSBCL had BCOR mutations or deletions, 2% had BRAF G469A mutation, and no BRAF V600E mutation.^[10]^

Thus, an accurate diagnosis of HCL or other diseases similar to the above can be established using complete immunophenotypes and molecular markers (Table 1).

**Table 1:** The differential diagnosis of SMZL, SDRPSBCL, HCL, HCL-V.

	Flow cytometry immunophenotype					
	CD11c	CD25	CD123	CD103	Bone marrow pathology immunochemistry	Genetic mutations
SMZL	+/−	−	−	−	BRAFV600E−	BRAFV600E−
SDRPSBCL	+	−	−	−	BRAFV600E−	BRAFV600E−
HCL	+	+	+	+	BRAFV600E+	BRAFV600E+
HCLv	+	−	−	+/−	BRAFV600E−	BRAFV600E−

HCL = hair cell leukemia, HCL-V = variant hair cell leukemia, SMZL = splenic marginal zone lymphoma, SDRPSBCL = diffuse splenic red myeloid small B-cell lymphoma

The patient we reported with classical HCL showed atypical immunophenotype (CD103 negative and CD23 positive) on both flow cytometry tests. However, bone marrow pathology and gene tests were positive for BRAF V600E, which supported the diagnosis of classical HCL. In classical HCL, the positive rate of CD23 ranged from 17% to 62.5%,^[6,9,12]^ and CD103 (−) rarely appeared in classical HCL. Chen et al found CD103 (−) in only 2 patients by flow cytometry in 35 cases of classical HCL^[12]^, among which 1 patient was accompanied by CD10 (+) and none by CD23 (+). In the largest series of cases reported by Yanying et al,^[6]^ no CD103 (−) cases were detected in 25 classical HCL cases in China. Shao et al reported that 169 classical HCL patients were all CD103 (−).^[7,9]^ Therefore, bone marrow pathological immunohistochemistry and genetic testing for BRAF V600E should be performed for the accurate diagnosis of classical HCL in cases with atypical immunophenotypes, especially CD103 (−) cases.

Patients with asymptomatic HCL can be observed temporarily without treatment. For HCL that requires treatment, purine nucleoside analogs such as cladribine or pentostatin monotherapy have been the standard first-line treatment for HCL for 30 years.^[1]^ Although complete response (CR) rates are high, ranging from 80% to 90%, there is often a positive MRD that leads to relapse and repeated treatment. MRD can be cleared by combination therapy with rituximab. Cladribine followed by rituximab (1 month after cladribine) showed 100% CR rates for both initial treatment HCL and relapsed HCL, with 5-year failure-free survival rates of 95% and 100%, respectively. Seventy-six percent of the initial treated HCL patients had negative MRD, and 64% of relapsed HCL patients had negative MRD.^[13]^ However, the optimal timing for rituximab is unknown. A randomized controlled phase 2 study found that nearly all patients (97%) receiving cladribine with concurrent rituximab achieved CR with negative MRD, which was significantly better than cladribine alone (24%). After a median follow-up of 96 months, 94% of patients in the cladribine with concurrent rituximab treatment group still maintained negative MRD CR, whereas the negative MRD CR rate in the cladribine delayed (≥6 months after clatribin) rituximab group was low (67%).^[14]^ Whether negative MRD CR means that HCL patients do not require additional treatment or cure still requires long-term follow-up monitoring. For this patient, we chose cladribine with concurrent rituximab treatment and achieved MRD (−) CR 1 month later.

## 4 Conclusions

Classical HCL has no specific immunophenotypes. In addition to clinical features and morphological characteristics of cells, complete immunophenotypic analysis (including CD11C, CD25, CD103, and CD123) and molecular detection (BRAFV600E mutation) can be differentiated from other HCL-like diseases and achieve accurate diagnosis. A combination of purine nucleoside analogs and rituximab resulted in a higher MRD (−) complete response rate.

## Author contributions

Contribution: Conceptualization by Hong-li Zhao, Hong-hua Cui, Wei-zhang Shen; Investigation by Hong-li Zhao, Hong-hua Cui, Li-fang Jin, Meng Zhao, Wei-zhang Shen; Writing–original draft by Hong-li Zhao, Hong-hua Cui, Li-fang Jin, Meng Zhao, Wei -zhang Shen; Writing–review and editing by Hong-li Zhao and Wei-zhang Shen.

**Conceptualization:** Hong-li Zhao, Hong-hua Cui, Li-fang Jin, Meng Zhao, Wei-zhang Shen.

**Investigation:** Hong-li Zhao, Hong-hua Cui, Li-fang Jin, Meng Zhao, Wei-zhang Shen.

**Writing – original draft:** Hong-li Zhao, Hong-hua Cui, Li-fang Jin, Meng Zhao, Wei-zhang Shen.

**Writing – review & editing:** Wei-zhang Shen.
