# CD103-CD23+ classical hairy cell leukemia: A case report and review of the literature: Erratum

**DOI:** 10.1097/MD.0000000000032565

**Published:** 2022-12-23

In the article, “CD103-CD23+ classical hairy cell leukemia: A case report and review of the literature,”^[1]^ the title has been corrected from “CD103-CD23+ classical hair cell leukemia: A case report and review of the literature.”

## References

[1] Zhao H-l Cui H-h Jin L-f. CD103-CD23+ classical hairy cell leukemia: a case report and review of the literature. Medicine (Baltimore). 100:e28262. 10.1097/MD.0000000000028262 PMC8702008 34941102

